# CircRNA_2646 functions as a ceRNA to promote progression of esophageal squamous cell carcinoma via inhibiting miR-124/PLP2 signaling pathway

**DOI:** 10.1038/s41420-021-00461-9

**Published:** 2021-05-11

**Authors:** Bo Zeng, Zhenguo Liu, Haoshuai Zhu, Xin Zhang, Weixiong Yang, Xiaoxing Li, Chao Cheng

**Affiliations:** 1 Department of Thoracic Surgery, the First Affiliated Hospital, Sun Yat-sen University, 510080 Guangzhou, China; 2 Institute of Precision Medicine, the First Affiliated Hospital, Sun Yat-sen University, 510080 Guangzhou, China

**Keywords:** Oesophageal cancer, Cell invasion

## Abstract

MicroRNA-124 (miR-124) has been predicted as a tumor suppressor in esophageal squamous cell carcinoma (ESCC). However, factors contributing to miR-124 reduction remain unclear. Circular RNAs (circRNAs) are a new family of non-coding RNAs with gene regulatory potential via interacting with miRNAs. We predicted three circRNAs, including CircRNA_14359, CircRNA_2646, and CircRNA_129, that could interact with miR-124 by bioinformatics analysis and determined their expressions in ESCC tissues and adjacent normal tissues. We found that CircRNA_2646 was up-regulated in ESCC, negatively correlated with the expression of miR-124 and positively associated with TNM stage and lymph node metastasis of ESCC. Luciferase reporter assay showed that CircRNA_2646 interacted with miR-124 in ESCC Eca109 and TE-1 cells. Moreover, ectopical overexpression of CircRNA_2646 accelerated cell proliferation, migration, invasion, and epithelial-to-mesenchymal transition (EMT), but restoration of miR-124 abrogated these functions and promoted Bcl-2-dependent cell apoptosis. Furthermore, it was found that the oncogene Proteolipid Protein 2 (PLP2) was the target gene of miR-124. In Eca109 and TE-1 cells, restoration of miR-124 decreased the level of PLP2 and inhibited PLP2-induced cell proliferation, migration, invasion, and EMT, but enhanced cell apoptosis. The in vivo study confirmed that CircRNA_2646 promoted ESCC development by repressing miR-124 and activating PLP2. Taken together, we identified that CircRNA_2646 functioned as an inhibitor in miR-124 signaling pathway in ESCC for carcinogenesis and could be a promising target for ESCC therapy.

## Introduction

Esophageal cancer frequently happens in the digestive system^1^. As the main type of esophageal cancer, esophageal squamous cell carcinoma (ESCC) accounts for 87% of all cases^2^. Due to the insidious symptoms, late clinical presentation, and rapid progression, the prognosis of ESCC remains extremely poor, with a 5-year survival rate of only 30–40% in patients who underwent surgery^3,4^. Therefore, a better understanding of the key molecular mechanisms of the disease is urgently needed.

Non-coding RNAs (ncRNAs) are endogenous RNA transcripts without protein-coding potential. Recently, several kinds of ncRNAs, including microRNAs (miRNAs), long noncoding RNAs (lncRNAs), and circular RNAs (circRNAs) have received extensive attention in various pathological conditions, such as cardiovascular diseases^5^, neurodegenerative diseases^6^, infection^7^, and cancers^8^. MiRNAs have only 21–25 nucleotides, and usually, negatively regulate gene expression at the post-transcriptional level by incompletely or completely binding to the 3′-untanslated regions (3′UTR) of target mRNAs^9^. Many miRNAs target oncogenes or tumor suppressors and thereby exert regulatory functions in the pathogenesis of various kinds of cancers^10,11^, including ESCC^12^. MiR-124, a well-known tumor-suppressive miRNA, was found significantly down-regulated in ESCC tissues^13^. We have reported that the low-expression of miR-124 was significantly correlated with the growth and invasion of ESCC via up-regulating branched-chain amino acid transaminase 1 (BCAT1)^14^. However, the mechanism of miR-124 in modulating ESCC progression by other pathways remains to be revealed.

CircRNAs are an abundant type of ncRNAs that shape a covalently closed continuous loop with no 5′–3′ polarity and without a poly-A tail, which makes them resistant to regular mechanisms of linear RNA decay and may serve as promising biomarkers and targets in cancer diagnosis and treatment^15^. Accumulating evidence demonstrated that circRNAs can bind to specific miRNAs and work as miRNA sponges and/or transporters to function as a critical regulator of oncogenes and tumor suppressors in ESCC^16,17^. It was reported that miR-124 was sponged by several circRNAs in cancer cells. For example, CircRNA-HIPK3 was found to regulate hepatocellular carcinoma proliferation and migration through sponging miR-124 and thereby modulating aquaporin 3^18^. CircRNA-MMP9 was demonstrated to act as the sponge of miR-124 and accelerate tumorigenesis of glioblastoma multiforme^19^. However, whether there are other circRNAs that join in the regulation of miR-124 remains unknown.

Proteolipid Protein 2 (*PLP2*) has been reported as an oncogene in several cancers, such as human gliomas^20^, malignant melanoma^21^, clear cell renal cell carcinoma^22^, breast cancer^23^, etc. The overexpression of PLP2 could facilitate the oncogenesis and proliferation of breast cancer^23^. It also acted as an important downstream target of miRNAs in tumor progression^21,22^. We found that PLP2 was a target gene of miR-124 and was upregulated in ESCC tissues and cell lines. However, the functions of PLP2 in ESCC have not been reported before.

Consider the novel function of circRNAs in cancer progression, we proposed that circRNAs could mediate the regulation of miR-124 in ESCC. In the present study, the interaction of CircRNA_2646 and miR-124 was identified by bioinformatics analysis, Furthermore, we found that miR-124 could mediate the proliferation, migration, invasion, and EMT of ESCC by targeting PLP2. The mechanisms of CircRNA_2646/miR-124/PLP2 axis in ESCC were explored in vitro and in vivo. This study will provide evidence for targeting the CircRNA_2646/miR-124/PLP2 axis for cancer therapy.

## Results

### Three predicted circRNAs targeting miR-124 in ESCC

**Fig. 1:**
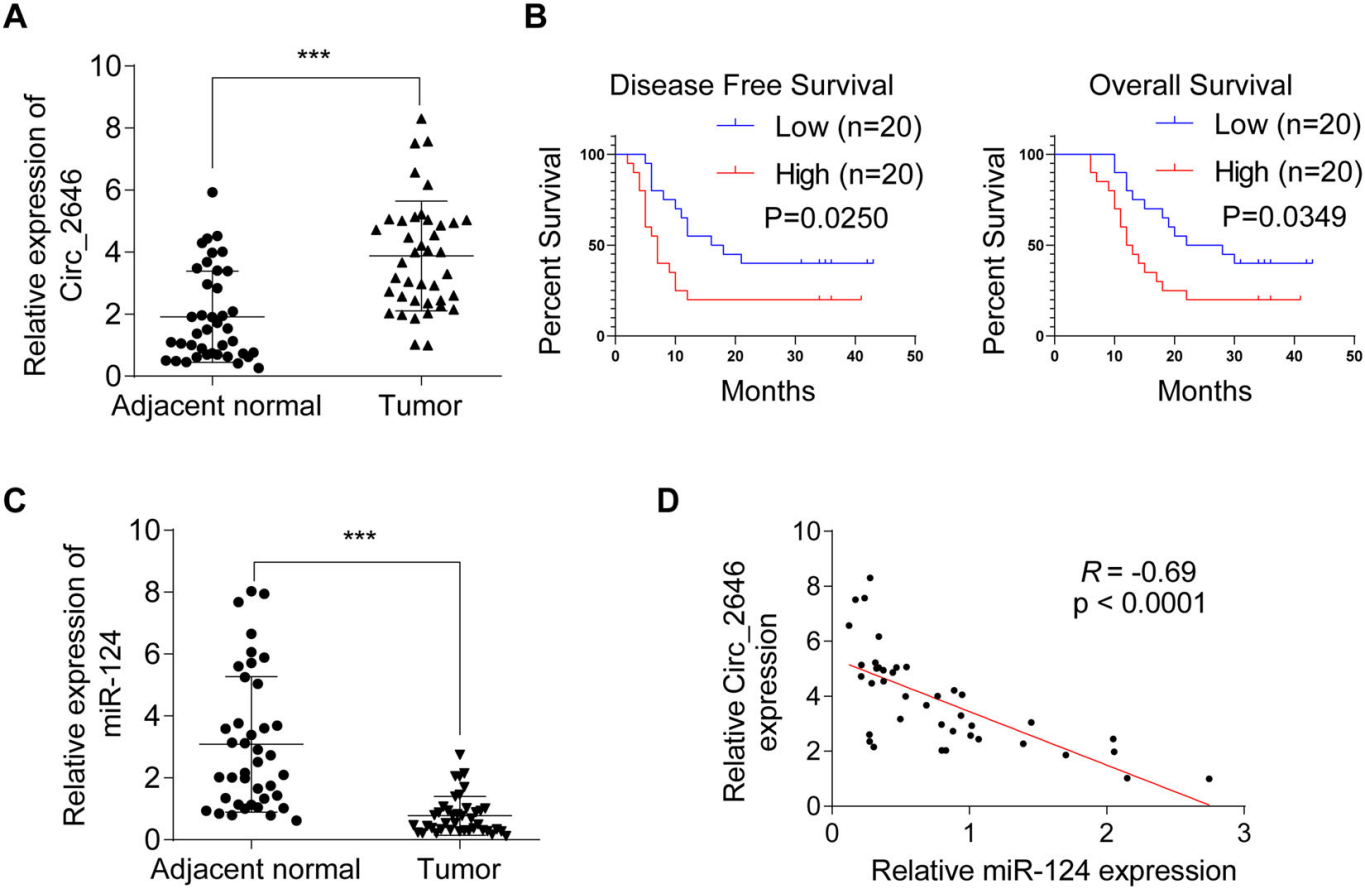
Three predicted circRNAs targeting miR-124 in ESCC. **A** The expression of CircRNA_2646 in ESCC tissues and adjacent normal tissues (*n* = 40) determined by RT-qPCR. *p* < 0.0001. **B** The correlation of the CircRNA_2646 expression and disease-free survival and overall survival of patients. *p* < 0.05. **C** The expression of miR-124 in ESCC tissues and adjacent normal tissues determined by RT-qPCR. **D** Correlation of CircRNA-2646 expression and miR-124 expression in ESCC tissues.

To investigate the potential regulatory circRNAs targeting miR-124, we utilized bioinformatics analysis (http://starbase.sysu.edu.cn) and predicted three circRNAs that harbored potential interacting sites of miR-124, including has-CircRNA_14359, has-CircRNA_2646, and has-CircRNA_129. The expressions of these three circRNAs in ESCC tissues and adjacent normal tissues were measured. Results showed that only CircRNA_2646 was remarkably upregulated in ESCC tissues (Fig. 1A, *p* < 0.0001). The expressions of the other two circRNAs were comparable in ESCC tissues and adjacent normal tissues (Supplementary Fig. 1A, B). By analyzing the correlation between CircRNA_2646 expression and clinicopathological features in ESCC patients, we found that patients with high CircRNA-2646 levels showed poor differentiation (Table 1, *p* < 0.05), high pathologic stage, and N stage (Table 1, *p* < 0.01). However, there was no significant difference between high and low CircRNA-2646 expression on patients’ age, gender, smoking status, and T stage. In addition, we found that high CircRNA_2646 expression was noticeably associated with the shorter disease-free survival and overall survival of patients (Fig. 1B, *p* < 0.05). These results indicated that CircRNA_2646 exerted an oncogenic role in ESCC and hinted that CircRNA_2646 might be the potential regulator of miR-124 in ESCC.

**Table 1:** Correlation between circRNA_2646 expression and clinicopathological characteristics in ESCC patients (*n* = 40).

clinicopathological characteristics	No. of patients	No. of patents with high expression of circRNA_2646 (*n*%)	*p* value
*Age (years)*			
<59	18	10 (55.6%)	0.376
≥59	22	10 (45.5%)	
*Gender*			0.642
Male	30	15 (50.0%)	
Female	10	5 (50.0%)	
*Smoking*			0.053
Yes	16	11 (68.8%)	
No	24	9 (37.5%)	
*Differentiation*			0.015*
Well	5	0 (0.00%)	
Moderate	20	10 (50.0%)	
Poor	15	10 (66.7%)	
*T stage*			0.367
1	9	4 (44.4%)	
2	30	15 (50.0%)	
3	1	1 (100.0%)	
*N stage*			0.002*
0	23	7 (30.4%)	
1	13	9 (69.2%)	
2	3	3 (100.0%)	
3	1	1 (100.0%)	
*TNM stage*			0.001*
I	1	0 (0.00%)	
II	21	6 (28.6%)	
III	17	13 (76.5%)	
VI	1	1 (100.0%)	

TNM stage: pathologic tumor, node, metastasis stage.

*p values < 0.05.

### Expression of CircRNA_2646 negatively correlated with miR-124 in ESCC

We next detected the expression of miR-124 in ESCC tissues and adjacent normal tissues and confirmed that miR-124 had low expression in ESCC tissues (Fig. 1C, *p* < 0.0001). Analyzing the correlation between miR-124 and CircRNA_2646, CircRNA_14359, or CircRNA_129 in ESCC tissues, we found that only CircRNA_2646 was negatively associated with the expression of miR-124 in ESCC tissues (Fig. 1D, *p* < 0.0001), implicating the regulatory role of CircRNA_2646 on miR-124in ESCC. This result indicated that CircRNA_2646 might promote the progression of ESCC via inhibiting miR-124.

### CircRNA_2646 interacted with miR-124 in ESCC cells

**Fig. 2:**
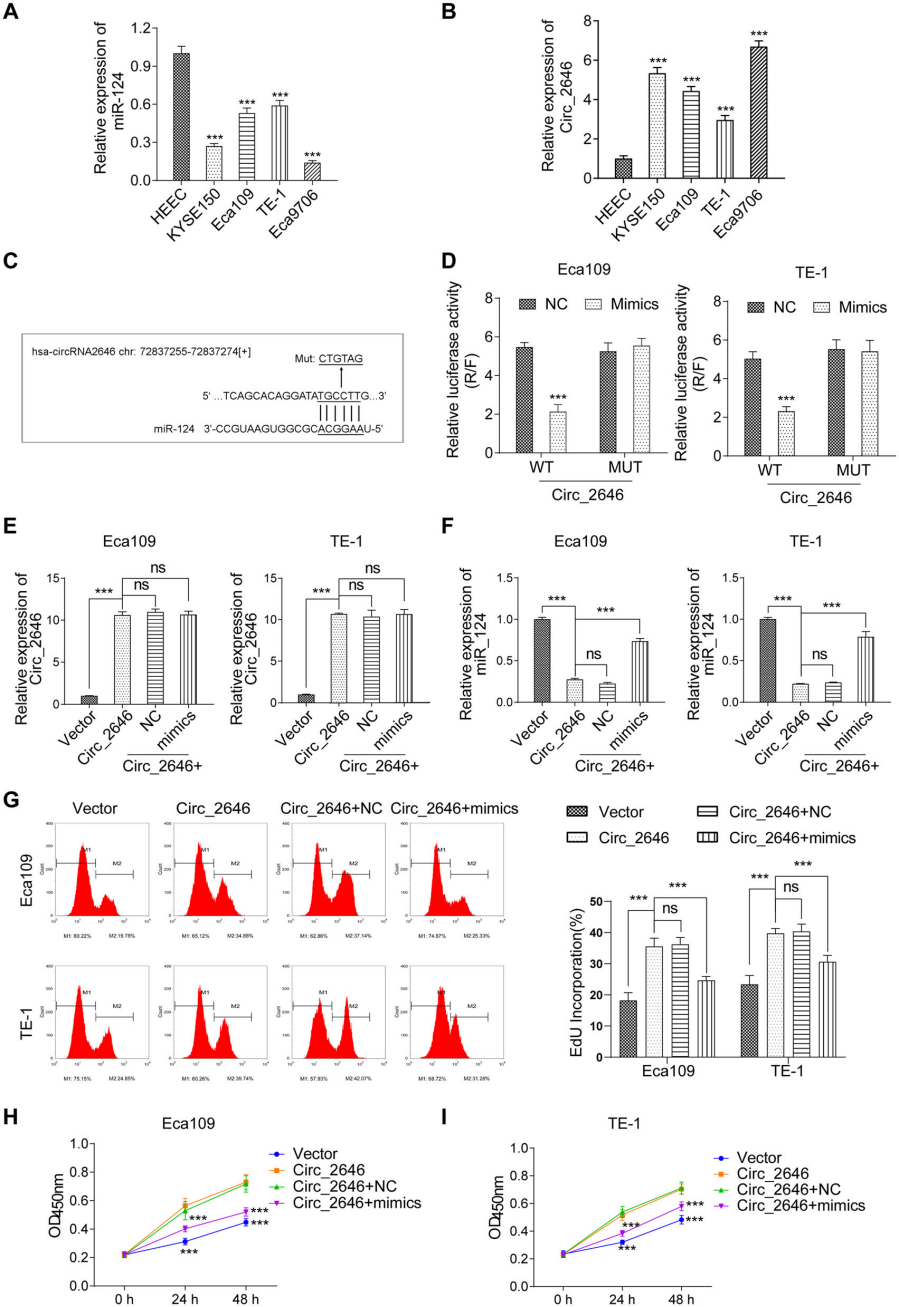
CircRNA_2646 interacted with miR-124 in ESCC cells. **A** and **B** The expressions of miR-124 and CircRNA_2646 determined by RT-qPCR in human normal esophageal epithelial cells HEEC and ESCC cell lines (KYSE150, Eca109, TE-1, and Eca9706). **C** CircRNA_2646 was predicted to harbor the potential interaction sites of miR-124 by bioinformatics analysis. **D** Luciferase reporter assay indicated that CircRNA_2646 could interact with miR-124 in Eca109 and TE-1 cells. **E** and **F** The expressions of CircRNA_2646 and miR-124 in Eca109 and TE-1 cells estimated by RT-qPCR after overexpression of CircRNA_2646 with or without miR-124 mimics transfection. **G** The proliferation and **H**, **I** viability of Eca109 and TE-1 cells determined by Edu and CCK-8 assay after relevant stimulation. ***p < 0.01.

The relationship between CircRNA_2646 and miR-124 was investigated in ESCC cells. We examined the expressions of miR-124 and CircRNA_2646 in human normal esophageal epithelial cells HEEC and ESCC cell lines (KYSE150, Eca109, TE-1, and Eca9706). Results in Fig. 2A and B showed that miR-124 was down-regulated in ESCC cell lines, but CircRNA_2646 was up-regulated in ESCC cells, compared to HEEC cells (*p* < 0.01). Because the expressions of miR-124 in Eca109 and TE-1 cells were higher than others, these two cell lines were selected for subsequent study. Luciferase reporter vectors of psiCHECK2-CircRNA_2646 with WT or MUT miR-124-binding sites (Fig. 2C) were designed to provide direct evidence of the interaction between CircRNA_2646 and miR-124. psiCHECK2-CircRNA_2646-WT or psiCHECK2-CircRNA_2646-MUT was co-transfected with miR-124 mimics into Eca109 and TE-1 cells. The results of luciferase reporter assays showed that miR-124 mimics transfection remarkably impaired the luciferase activity of psiCHECK2-CircRNA_2646-WT (Fig. 2D, *p* < 0.01), but had no significant effect on the luciferase activity of psiCHECK2-CircRNA_2646-MUT in Eca109 and TE-1 cells. These data illustrated that CircRNA_2646 could exert its regulatory function on the progression of ESCC via interacting with miR-124.

### CircRNA_2646 promoted ESCC cell growth and metastasis via inhibiting miR-124

**Fig. 3:**
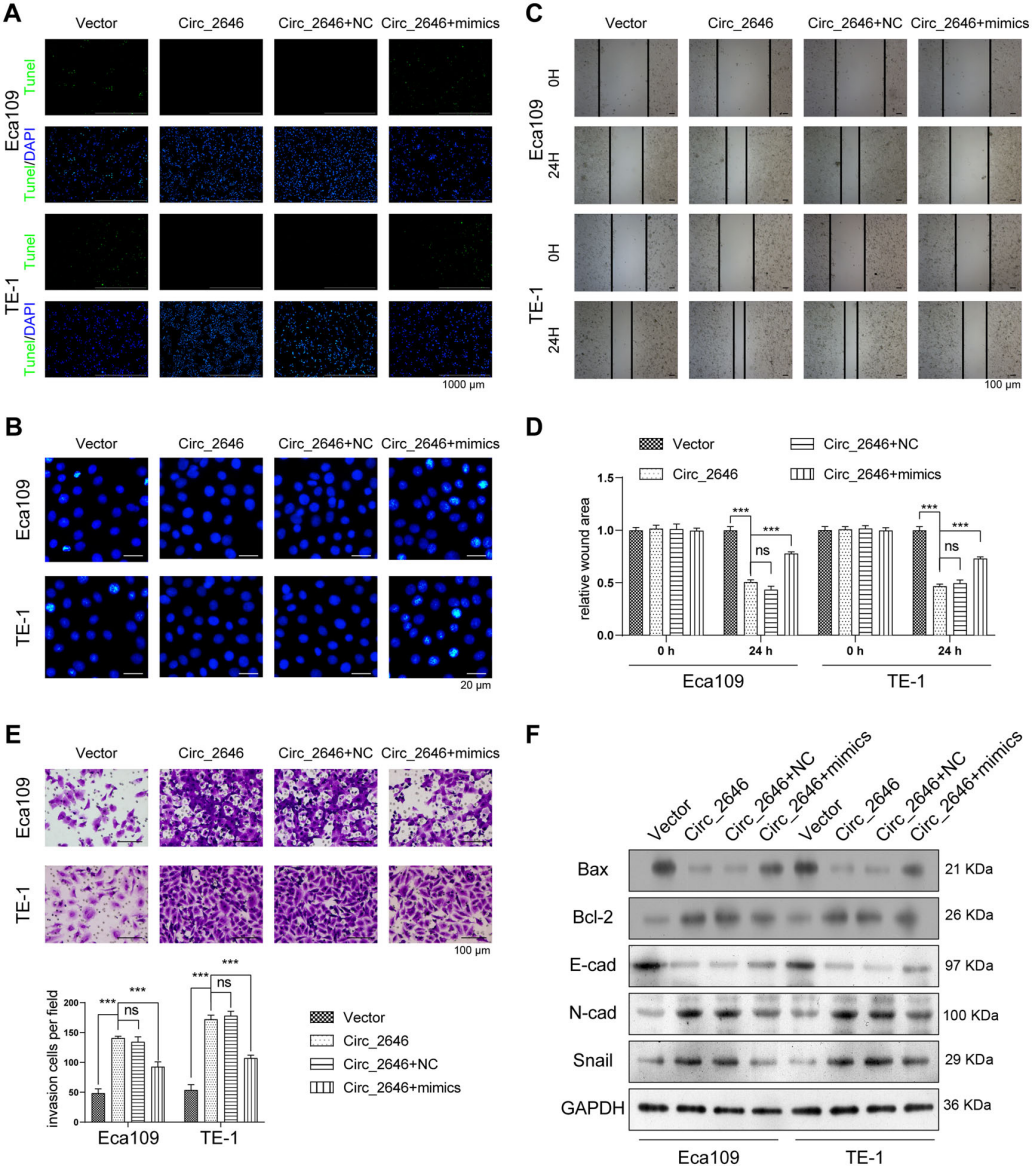
CircRNA_2646 inhibited ESCC cell apoptosis and promoted metastasis via inhibiting miR-124. After overexpression of circRNA_2646 with or without miR-124 mimics transfection, the apoptosis of Eca109 and TE-1 cells were determined by **A** Hoechst staining, scale bars, 1000 μm. **B** TUNEL assay, scale bars, 20 μm. The ability of migration and invasion estimated by **C**, **D** scratch wound assay, scale bars, 100 μm. **E** transwell assay, scale bars, 100 μm. **F** The key proteins in apoptosis pathway and EMT pathway analyzed by Western Blot. ***p < 0.01.

Subsequently, we investigated the function of CircRNA_2646 overexpression and miR-124 overexpression on Eca109 and TE-1 cell proliferation, apoptosis, migration, invasion, and EMT process. Figure 2E and F showed that single CircRNA_2646 transfection significantly increased CircRNA_2646 expression in Eca109 and TE-1 cells (*p* < 0.01), but obviously inhibited miR-124 level (*p* < 0.01). Moreover, miR-124 mimics had no influence on CircRNA_2646 expression but weakened the effects of CircRNA_2646 on miR-124 expression in Eca109 and TE-1 cells (*p* < 0.01). Results of the Edu assay and CCK-8 assay displayed that CircRNA_2646 promoted proliferation and elevated the viability (Fig. 2G–I, *p* < 0.01), while miR-124 mimics notably weakened the effects of CircRNA_2646 on proliferation and viability of Eca109 and TE-1 cells (*p* < 0.01). In addition, results of apoptosis assay showed that miR-124 mimics transfection-induced apoptosis of Eca109 and TE-1 cells, although these cells were forced to express CircRNA_2646 (Fig. 3A and B). In addition, CircRNA_2646 also enhanced the ability of migration and invasion in Eca109 and TE-1 cells via inhibition of miR-124 (Fig. 3C–E, *p* < 0.01). The expression of apoptosis-related proteins Bax and Bcl-2, as well as the EMT-related proteins E-cad, N-cad, and Snail, were estimated. Results in Fig. 3F displayed that the level of anti-apoptosis protein Bcl-2 was increased by CircRNA_2646 but reduced by miR-124 mimics. The level of pro-apoptosis protein Bax showed the opposite result. Furthermore, the level of anti-EMT protein E-cad was decreased by CircRNA_2646 but was increased by miR-124 mimics. The pro-EMT proteins N-cad and Snail levels showed the opposite tendency. These data illustrated that circRNA-2646 could promote ESCC cell growth and metastasis via inhibiting miR-124.

### Oncogene PLP2 was the target gene of miR-124 in ESCC

**Fig. 4:**
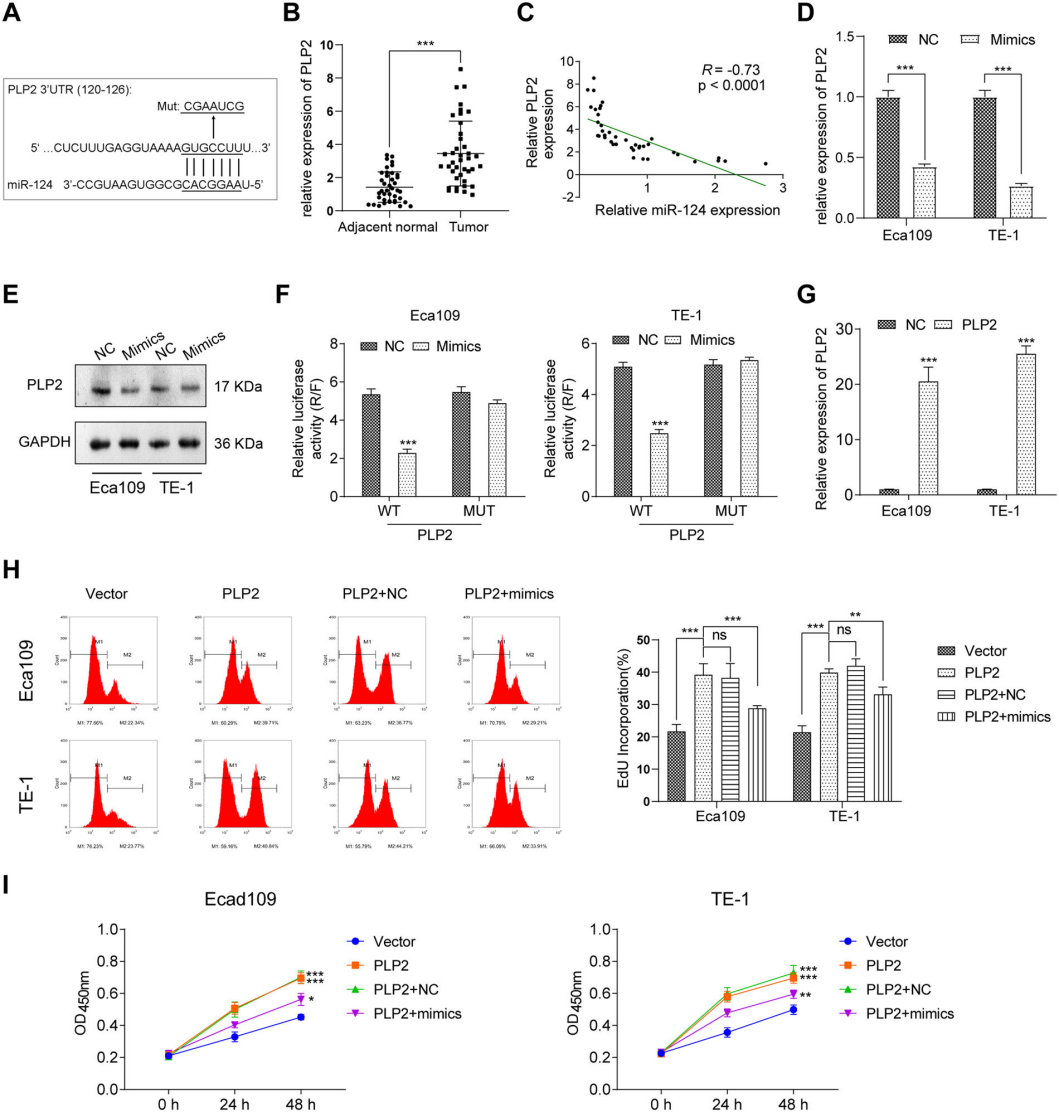
Oncogene PLP2 was the target gene of miR-124 and promoted ESCC cell growth. **A** PLP2 was predicted to harbor the potential interaction sites of miR-124 by bioinformatics analysis. **B** The expression of PLP2 in ESCC tissues and adjacent normal tissues was determined by RT-qPCR. **C** The correlation of the miR-124 expression and PLP2 expression in ESCC tissues. **D**, **E** Overexpression of miR-124 inhibited the mRNA and protein expressions of PLP2 in Eca109 and TE-1 cells. **F** Luciferase reporter assay indicated that PLP2 was a target gene of miR-124. **G** The PLP2 expression in Eca109 and TE-1 cells after PLP2 overexpression. **H** The proliferation and **I** viability of Eca109 and TE-1 cells determined by Edu assay and CCK-8 assay. ***p < 0.01.

The downstream target gene of CircRNA_2646/miR-124 axis needed to be further explored. Bioinformatics analysis with http://www.targetscan.org/vert_71/ indicated that the 3′UTR of oncogene PLP2 mRNA had the binding site of miR-124 (Fig. 4A). Moreover, PLP2 was upregulated in ESCC tissues compared to adjacent normal tissues (Fig. 4B, *p* < 0.001). PLP2 expression was negatively related to miR-124 expression in ESCC tissues (Fig. 4C, *p* < 0.001). In addition, miR-124 mimics obviously inhibited the mRNA and protein levels of PLP2 in Eca109 and TE-1 cells (Fig. 4D, E, *p* < 0.01 in mRNA level). The results of luciferase reporter assay showed that miR-124 mimics significantly impaired the luciferase activity of psiCHECK2-GPRC5A-WT (Fig. 4F, *p* < 0.01). These results further demonstrated that PLP2 was a target gene of miR-124 in ESCC cells.

### Restoration of miR-124 inhibited PLP2-induced ESCC cell growth and metastasis

**Fig. 5:**
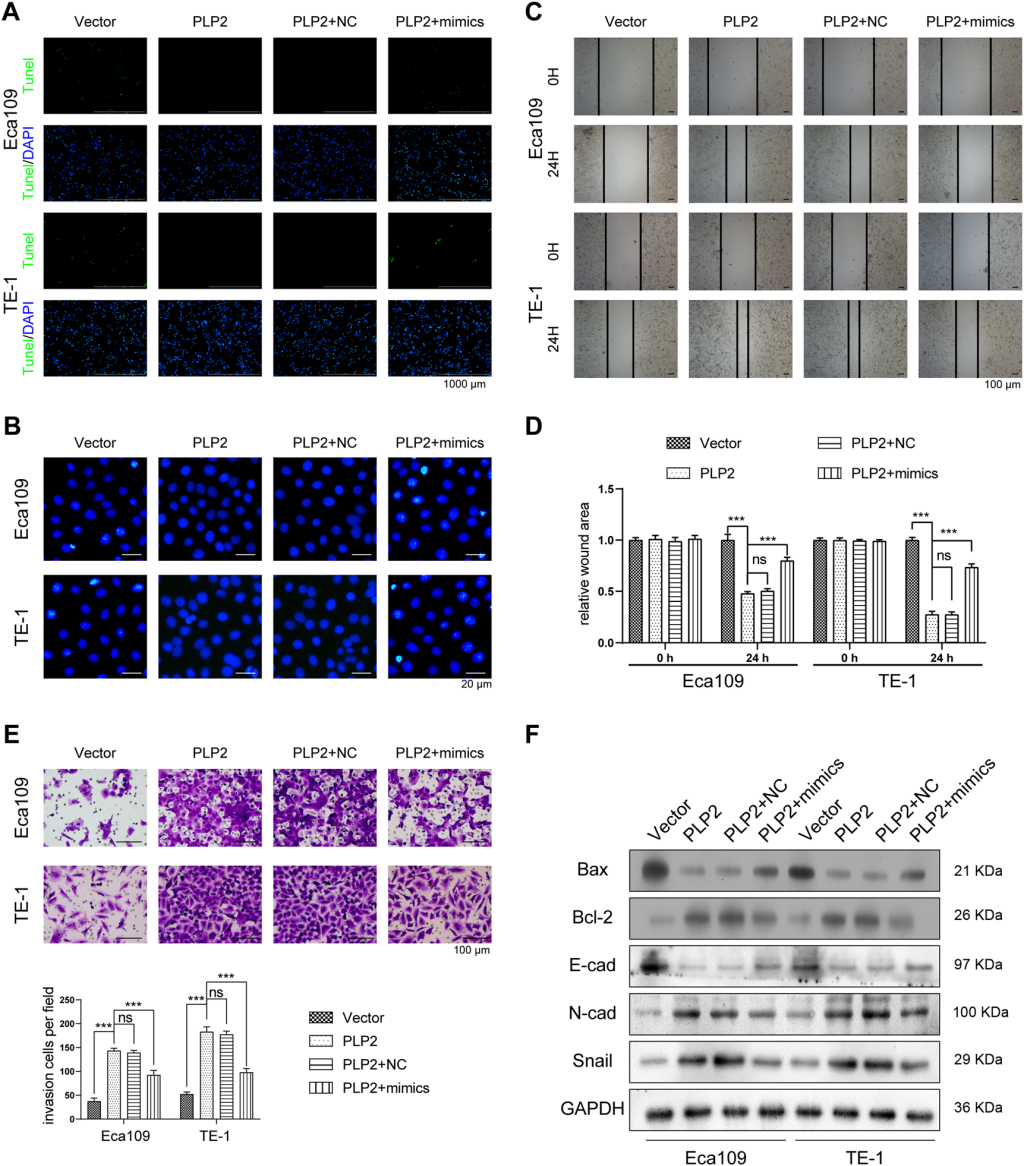
Restoration of miR-124 weakened PLP2-caused ESCC cell metastasis and promoted cell apoptosis. After overexpression of PLP2 with or without miR-124 mimics transfection, the apoptosis of Eca109 and TE-1 cells was determined by **A** Hoechst staining, scale bars, 1000 μm. **B** TUNEL assay, scale bars, 20 μm. The ability of migration and invasion was estimated by **C**, **D** scratch wound assay, scale bars, 100 μm. **E** transwell assay, scale bars, 100 μm. **F** The key proteins in apoptosis pathway and EMT pathway analyzed by WB. **p < 0.01.

**Fig. 6:**
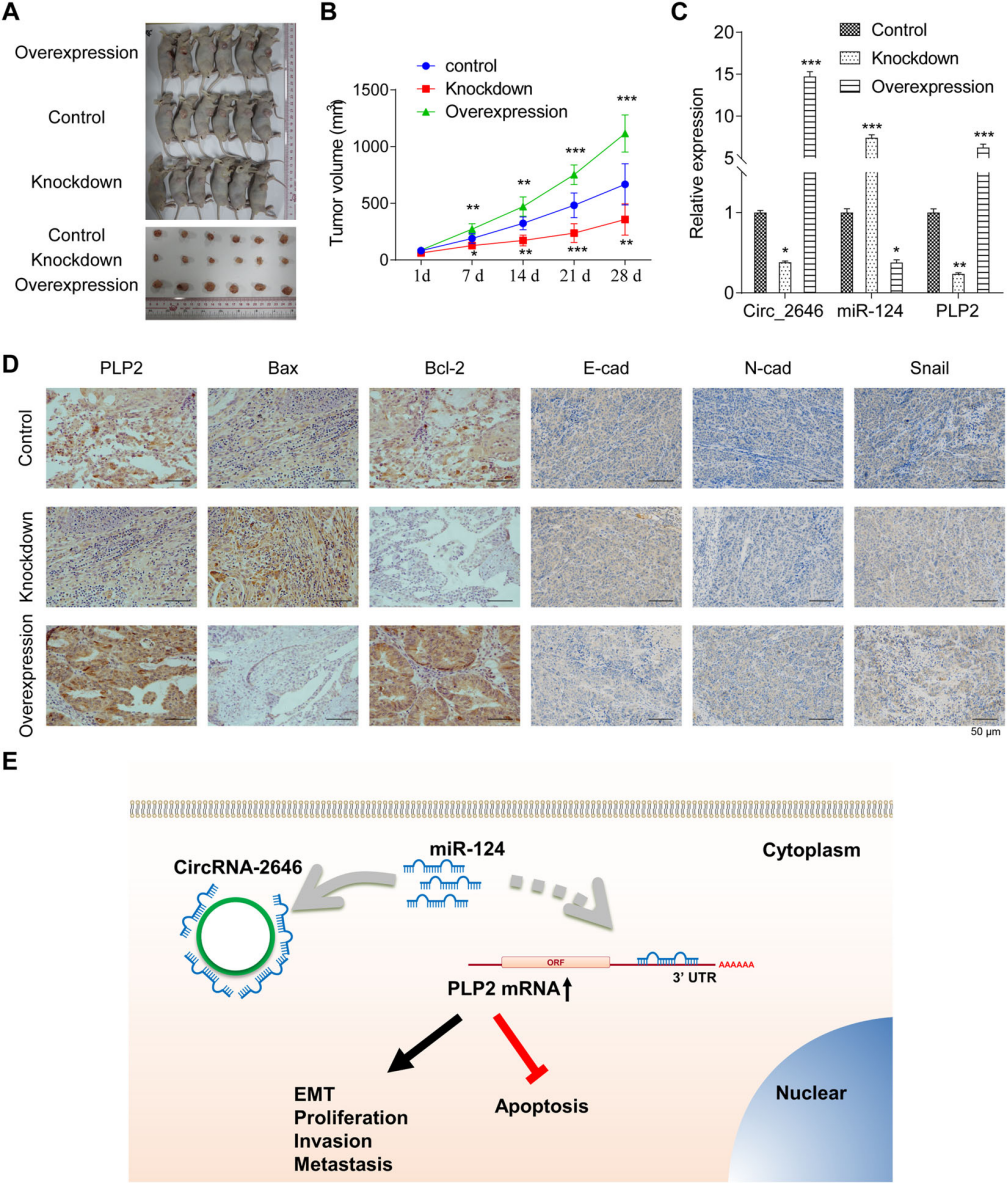
CircRNA_2646 promoted ESCC development in vivo via regulating miR-124/PLP2. Eca109 cells infected with Lentivirus-pcDNA3.1-circRNA_2646, shRNA-circRNA_2646, or negative control were subcutaneously injected into the right flank of nude mice. **A** The picture of the final tumor from each group. **B** The tumor volume of each group was calculated and analyzed. **C** The expressions of CircRNA_2646, miR-124, and PLP2 in tumor tissues were analyzed by RT-qPCR. **D** The expressions of PLP2, Bax, Bcl-2, E-cad, N-cad, and Snail in tumor tissues were determined by IHC, scale bar, 50 μm. *p < 0.05, **p < 0.01, ***p < 0.001. **E** The proposed pathway of CircRNA-2646/miR-124/PLP2 axis on ESCC progression. CircRNA-2646 functions as an inhibitor of the miR-124/PLP2 signaling pathway to enhance ESCC cell proliferation, migration, invasion, and EMT progress, and inhibit cell apoptosis.

We further explored the function of miR-124 and PLP2 on ESCC cells. PLP2 was transfected into Eca109 and TE-1 cells to increase PLP2 expression (Fig. 4G, *p* < 0.01). The results of the Edu assay and CCK-8 assay showed that PLP2 promoted proliferation and increased cell viability of Eca109 and TE-1 cells (Fig. 6H and I, *p* < 0.01). MiR-124 mimics weakened the effects of PLP2 on proliferation and viability of Eca109 and TE-1 cells (*p* < 0.01). Moreover, miR-124 mimics induced the apoptosis of Eca109 and TE-1 cells, although these cells were forced expressed PLP2 (Fig. 5A and B). Meanwhile, the migration and invasion ability of Eca109 and TE-1 cells were also promoted by PLP2 but were abrogated by miR-124 mimics (Fig. 5C–E, *p* < 0.01). Mechanistically, anti-apoptosis protein Bcl-2 expression was increased by PLP2 but was reduced by miR-124 mimics (Fig. 5F). The pro-apoptosis protein Bax expression showed the opposite result. Besides, PLP2 also reduced the expression of E-cad, but enhanced the expressions of N-cad and Snail to promote the EMT process of Eca109 and TE-1 cells. MiR-124 mimics inhibited the PLP2 expression to reduce the EMT process of Eca109 and TE-1 cells.

### CircRNA_2646 promoted ESCC development in vivo via regulating the miR-124/PLP2 pathway

The xenograft model of ESCC was established using Eca109 cells with CircRNA_2646 knockdown or overexpression to confirm the influence of CircRNA_2646 on ESCC development. The picture of final tumor tissues from each group was presented in Fig. 6A. Figure 6B showed that the mice with CircRNA_2646 knockdown had the smallest tumor volume (*p* < 0.01 or *p* < 0.001), accompanied by the increased miR-124 expression and decreased PLP2 expression in tissues (Fig. 6C, *p* < 0.01). CircRNA-2646 overexpression had the opposite effect (*p* < 0.05, *p* < 0.01, or *p* < 0.001). Analysis of the Bax, Bcl-2, E-cad, N-cad, and Snail expressions in tumor tissues by IHC staining showed that CircRNA_2646 overexpression lowered Bax and E-cad levels, but enhanced the Bcl-2, N-cad, and Snail levels in ESCC tissues of mice (Fig. 6D). CircRNA_2646 knockdown had the opposite effects. These results confirmed that CircRNA_2646 was a critical regulator of miR-124 and promoted ESCC development.

## Discussion

In this study, we first demonstrated that CircRNA_2646 was up-regulated in ESCC and its expression was negatively correlated with miR-124. Meanwhile, a high CircRNA_2646 level was significantly associated with several clinical-pathological characteristics and poor prognosis. Second, we showed that upregulation of CircRNA_2646 decreased the expression of miR-124 and promoted the malignant progression of ESCC. Third, CircRNA_2646 activated the PLP2 signaling pathway via sponging miR-124. Finally, our data revealed the function and potential of CircRNA_2646/miR-124/PLP2 axis in the development of ESCC.

As a novel type of ncRNAs, CircRNAs have been considered as promising biomarkers in cancers^24^. Clarifying the regulatory mechanism of circRNAs and miRNAs during tumorigenesis has become a hot spot in the field of molecular biology research^25^. Increasing literature demonstrated that CircRNAs, serving as sponges of miRNAs or RNA-binding protein (RBP)^26^, play important regulatory roles in biological processes or various tumors, including hepatocellular carcinoma^18^, glioblastoma^19^, and ESCC^27^. Some other circRNA–miRNA networks, such as CircRNA-100367–miR-217^16^, CircRNA–ciRS7–miR-7^28^, CircRNA-0006948–miR-490^29^, and circ-VRK1–miR-624^30^, were reported to play critical roles in the pathogenesis of pro-ESCC or anti-ESCC. In this study, we found that overexpression of CircRNA_2646 inhibited the expression of miR-124 and promoted the proliferation, migration, and invasive ability of ESCC cells via binding to the miR-124 sequence. These findings evidenced that CircRNA_2646 acted on the progression of ESCC through sponging miR-124.

PLP2 is an endoplasmic reticulum protein, which has been viewed as a tumor-promoting factor in various cancers, including glioma^20^, cutaneous malignant melanoma^21^, clear cell renal cell carcinoma^22^, and breast cancer^23^. So far, there is no literature on the role of PLP2 in the pathogenesis of ESCC. For the first time, we uncovered that PLP2 was a potential target gene of miR-124 and overexpressed in ESCC. The up-regulation of miR-124 reduced PLP2 expression in ESCC cells might be through binding to 3′UTR of the PLP2 mRNA. Functional experiments validated that overexpression of PLP2 could promote the proliferative, migration, and invasive abilities, as well as the EMT process of ESCC cells, while restoration of miR-124 inhibited the function of PLP2. These findings illustrated that PLP2 was a downstream factor of the CircRNA_2646/miR-124 axis in ESCC. Because PLP2 was modulated by other miRNAs in breast cancer^23^ and colorectal cancer^31^, it is not excluded that there are other circRNAs/miRNAs that might involve in the regulation of PLP2 in ESCC.

There are several limitations in this study. Firstly, the clinical samples were relatively small, which limited the stratified analysis to some extent. Secondly, although several molecular biology techniques were performed to delineate the functions of CircRNA_2646, miR-124, and PLP2 axis, more studies based on different cancers were needed to further investigate this regulatory axis. Therefore, further mechanistic research should be performed, and more patient samples need to be collected for further study in the future.

In summary, our study suggested that CircRNA_2646 promoted cell proliferation, migration, invasion, and EMT of ESCC cells by binding to miR-124. MiR-124 can affect the progression of ESCC by negatively regulating the expression of PLP2. Therefore, CircRNA_2646, miR-124, and PLP2 might be potential biomarkers in ESCC and could be promising therapeutic targets for cancer treatments.

## Material and methods

### Clinical specimens

Ethical approval for this study was obtained from the Ethics Committee of the First Affiliated Hospital, Sun Yat-sen University. All recruited patients were informed of this study and signed informed consent before surgery. ESCC tissues (*n* = 40) and adjacent normal tissues (>2 cm from tumor tissue) were collected from 2015 to 2016 in the Department of Thoracic Surgery. All patients diagnosed with primary ESCC were confirmed by experienced pathologists on hematoxylin and eosin staining slides. None of these patients received chemotherapy or radiotherapy before the study. Patients who were diagnosed with autoimmune or other malignant diseases and pregnant or lactating individuals were excluded from our experimental group. The clinical-pathological parameters and clinical features of all patients are shown in Table 1. All tissue specimens were stored in a liquid nitrogen container immediately after collection.

### Cell lines and reagents

Human normal esophageal epithelial cells HEEC and ESCC cell lines (KYSE150, Eca109, TE-1, and Eca9706) were obtained from ATCC, USA. The cell lines were maintained in a low-glucose DMEM culture medium containing 10% fetal bovine serum (FBS), 100 U/ml penicillin, and 100 μg/ml streptomycin with 5% CO_2_ under humidified condition. The pCD5-circRNA_2646, pcDNA3.1-PLP2, their negative control vectors, and the renilla luciferase reporter vector psiCHECK2-circRNA_2646 with wild (WT) or mutant (MUT) miR-124-binding sites were constructed by GenePharma (Shanghai, China). The oligonucleotide sequences of miR-124 mimics or negative control (NC) were purchased from RiboBio (Guangzhou, China). A reporter plasmid of full-length 3′-UTR (WT or MUT) of PLP2 was constructed by GenePharma (Shanghai, China).

### RNA isolation and real-time transcription PCR

Total RNA was isolated using Trizol reagent (Thermo Fisher Scientific, Waltham, MA, USA) according to the manufacturer’s instructions. cDNA was synthesized using the Bestar qPCR RT Kit (DBI Bioscience, Shanghai, China). Quantitative real-time RT-PCR (qRT-PCR) was performed using Bestar qPCR Master Mix (DBI Bioscience) on the ABI real-time PCR system (Applied Biosystem, USA). GAPDH and U6 were used as internal controls. The 2^−ΔΔCt^ method was applied to quantify the RNA expression. The primers are listed in Table 2.

**Table 2:** Primers for different genes.

Gene	Primer
hsa-miR-124-F	ACACTCCAGCTGGGTAAGGCACGCGGTGAATG
hsa-miR-124-R	TAAGGCACGCGGTGAATGCCAA
circRNA_14359-F	TCCTATTGCTCTTCCTTGTGGAAA
circRNA_14359-R	ACAGTTCGGAACCCACATCAA
CircRNA_2646-F	GGATGACAACACAGTTATAATCC
CircRNA_2646-R	AGCAAAGAGCTTCTCCAGGTT
circRNA_129-F	TTGCTGTATTTTTCCAGAATGCCT
circRNA_129-R	CCAACGAAAAGCCAAATGCG
U6-F	CTCGCTTCGGCAGCACA
U6-R	AACGCTTCACGAATTTGCGT
GAPDH-F	ACACCCACTCCTCCACCTTT
GAPDH-R	TTACTCCTTGGAGGCCATGT
PLP2-F	GCGCACTCGAAAGGGAATC
PLP2-R	AGGATCATCTCAATCACCGACA

### Cell transfection

CircRNA_2646, vector, miR-124 mimics, NC, and PLP2 were transfected into Eca109 and TE-1 cells by using Transfection 2000 reagent (Invitrogen, Carlsbad, CA, USA). After 48 h of transfection, the expressions of circRNA_2646, miR-124, and PLP2 were measured.

### Luciferase reporter assay

The Eca109 and TE-1 cells were co-transfected with firefly Luciferase vector, psiCHECK2–circRNA_2646–WT or MUT, psiCHECK2-3′UTR of PLP2 (WT-type or MUT type) and miR-124 mimics. Luciferase activity was measured using the Dual-Luciferase Reporter Assay System (Promega, Madison, WI, USA). Firefly luciferase acted as a reporter gene for normalized control. The activities of Firefly and Renilla luciferase were evaluated successively by applying a dual-luciferase reporter assay system (Promega) 48 h after transfection according to the manufacturer’s protocol.

### CCK-8 assay

After transfection, the viability of Eca109 and TE-1 cells was determined by the CCK-8 kit (Dojindo, Japan) at 0, 24, 48 h after transfection in 96-well plates. Each well was added with10 μl CCK-8 solution and incubated at 37 °C for 2 h. Light absorbance was detected using a microplate reader (Power Wave X; BioTek, Winooski, VT, USA) at 450 nm.

### EdU assay

After transfection, Eca109 and TE-1 cells were incubated with 100 μl of 50 μM EdU per well for 2 h at 37 °C and were fixed for 30 min at room temperature using 100 μl of fixing buffer (4% polyformaldehyde). Subsequently, the cells were incubated with 50 μl of 2 mg/ml glycine for 5 min followed by washing with 100 μl of phosphate buffer saline (PBS). The cells were treated with 0.5% Triton X and reacted with 1× Apollo solution for 30 min and then were incubated with 100 μl of 1× Hoechst solution for 30 min at room temperature in the dark followed by washing with 100 μl of PBS. Results were analyzed using flow cytometry.

### Western blotting

After transfection, Eca109 and TE-1cells were collected and the proteins were extracted. Then, the proteins were separated by sodium dodecyl sulfate–polyacrylamide gel electrophoresis (SDS–PAGE), transferred onto polyvinylidene fluoride (PVDF) membrane, and blocked in 5% non-fat milk in TBST buffer (Tris buffer saline containing 0.1% Tween-20) for 1 h at room temperature. Subsequently, the membranes were incubated with anti-PLP2, GAPDH, Bcl-2, Bax, Snail, E-cadherin (E-cad), and N-cadherin (N-cad) antibodies (Abcam. Cambridge, MA, USA) overnight at 4 °C. After washing with TBST buffer, the blots were then incubated with HRP-conjugated secondary antibody for 1 h at room temperature. The signals of blots were visualized using the ECL-Plus reagent (Millipore, Billerica, MA, USA). GAPDH was used as the loading control.

### Apoptosis assay

After transfection, the apoptosis of Eca109 and TE-1 cells was determined by Hoechst staining and TUNEL assay. For the Hoechst staining, cells were harvested and incubated with Hoechst 33342 (5 μg/ml, Sigma, USA) for 10 min at room temperature. Following washing with 0.5% Triton X-100 in PBS, the changes of nuclear morphology were observed under a fluorescence microscope (OLYMPUS, Tokyo, Japan). For TUNEL assay, Dead End™ Fluorometric TUNEL System (Promega, Madison, WI) was used according to the manufacturer’s instructions. The stained cells were examined under a fluorescence microscope.

### The scratch wound assay

The Eca109 and TE-1 cells were transfected with indicated reagents and wounded with a plastic tip that was dragged across the confluent cell monolayer. After 24 h, five fields were randomly selected and photographed under a light microscope (OLYMPUS, Tokyo, Japan).

### Transwell assay

The upper chamber of the transwell inserts was coated with 50 μl of 2.0 mg/ml Matrigel, 2 × 10^4^ Eca109, and TE-1 cells were plated in the upper chamber and the lower chamber contained 600 μl medium with 10% FBS to promote cell invasion. After 24 h of incubation, the non-invade cells in the upper chamber were removed using a cotton swab. Followed by crystal violet staining, the cells were counted under an inverted microscope. Five random views were selected, and the independent experiments were repeated three times.

### Xenograft tumor model

5-week-old male BALB/c nude mice were randomly divided into three groups (NC, overexpression, and knockdown groups). Each group composed of six mice that were injected with 2 × 10^6^ Eca109 cells with circRNA-2646 knockdown or overexpression. The tumor volume was calculated by measuring the length and width of the tumor (tumor volume = 1/2 length × width^2^) every week. After 4 weeks, the mice were sacrificed and the tumors were removed, weighed for analysis. All animal studies were approved by the Animal Ethics Committee of the First Affiliated Hospital, Sun Yat-sen University, and experiments were conducted according to the guidelines of the National Institutes of Health Guide for the Care and Use of Laboratory Animals.

### Immunohistochemistry (IHC) staining

Immunohistochemical staining for PLP2, Bcl-2, Bax, Snail, E-cad, and N-cad were performed on 5-μm-thick, formalin-fixed, and paraffin-embedded specimen sections as previously described^32^. Briefly, after de-paraffinizing in xylene solution and hydrating in gradient descent ethanol solution, the sections were placed in EDTA-antigen retrieval solution and boiled. Then, the sections were incubated with 3% H_2_O_2_ solution for 25 min, 3% bovine serum albumin (BSA) solution for 30 min, and primary antibodies overnight at 4 °C. The next day, sections were incubated with HRP-conjugated secondary antibodies for 1 h and developed in diaminobenzidine (DAB) solution. The sections were counterstained using hematoxylin solution. Finally, the coverslips were mounted onto the glass slides with neutral gum and observed under an FV10i microscope (OLYMPUS, Tokyo, Japan).

### Statistics

All studies were performed with three independent experiments. Statistical analyses were carried out using The Graphpad Prism (Version 8.0, Graphpad Software Inc.). Data were expressed as the mean ± standard deviation (mean ± SD). The Student’s *t*-test was used to analyze differences between two experimental groups. The differences among more than two groups were analyzed by one-way analysis of variance (ANOVA). Pearson correlation coefficients and two-tailed *p* values were calculated in analyzing the correlation of miRNA and CircRNAs. *p* values <0.05 were considered statistically significant.

## Supplementary information

primers for different genes

The expression of CircRNA_129 and CircRNA_14359
