# Supplementary material for: CircRNA_2646 functions as a ceRNA to promote progression of esophageal squamous cell carcinoma via inhibiting miR-124/PLP2 signaling pathway
Source: Cell Death Discov. 2021 May 11;7:99. doi: 10.1038/s41420-021-00461-9 (PMC8113544; doi:10.1038/s41420-021-00461-9)
Supplement: Supplementary file 1 — primers for different genes [file 41420_2021_461_MOESM1_ESM.docx]

**Supplemental Table.** primers for different genes

| **Gene** | **Primer** |
| --- | --- |
| hsa-miR-124-F | ACACTCCAGCTGGGTAAGGCACGCGGTGAATG |
| hsa-miR-124-R | TAAGGCACGCGGTGAATGCCAA |
| circRNA14359-F | TCCTATTGCTCTTCCTTGTGGAAA |
| circRNA14359-R | ACAGTTCGGAACCCACATCAA |
| CircRNA-2646-F | GGATGACAACACAGTTATAATCC |
| CircRNA-2646-R | AGCAAAGAGCTTCTCCAGGTT |
| circRNA129-F | TTGCTGTATTTTTCCAGAATGCCT |
| circRNA129-R | CCAACGAAAAGCCAAATGCG |
| U6-F | CTCGCTTCGGCAGCACA |
| U6-R | AACGCTTCACGAATTTGCGT |
| GAPDH-F | ACACCCACTCCTCCACCTTT |
| GAPDH-R | TTACTCCTTGGAGGCCATGT |
| PLP2-F | GCGCACTCGAAAGGGAATC |
| PLP2-R | AGGATCATCTCAATCACCGACA |
