# Supplementary figures and images for: CircRNA_2646 functions as a ceRNA to promote progression of esophageal squamous cell carcinoma via inhibiting miR-124/PLP2 signaling pathway
Source: Cell Death Discov. 2021 May 11;7:99. doi: 10.1038/s41420-021-00461-9 (PMC8113544; doi:10.1038/s41420-021-00461-9)

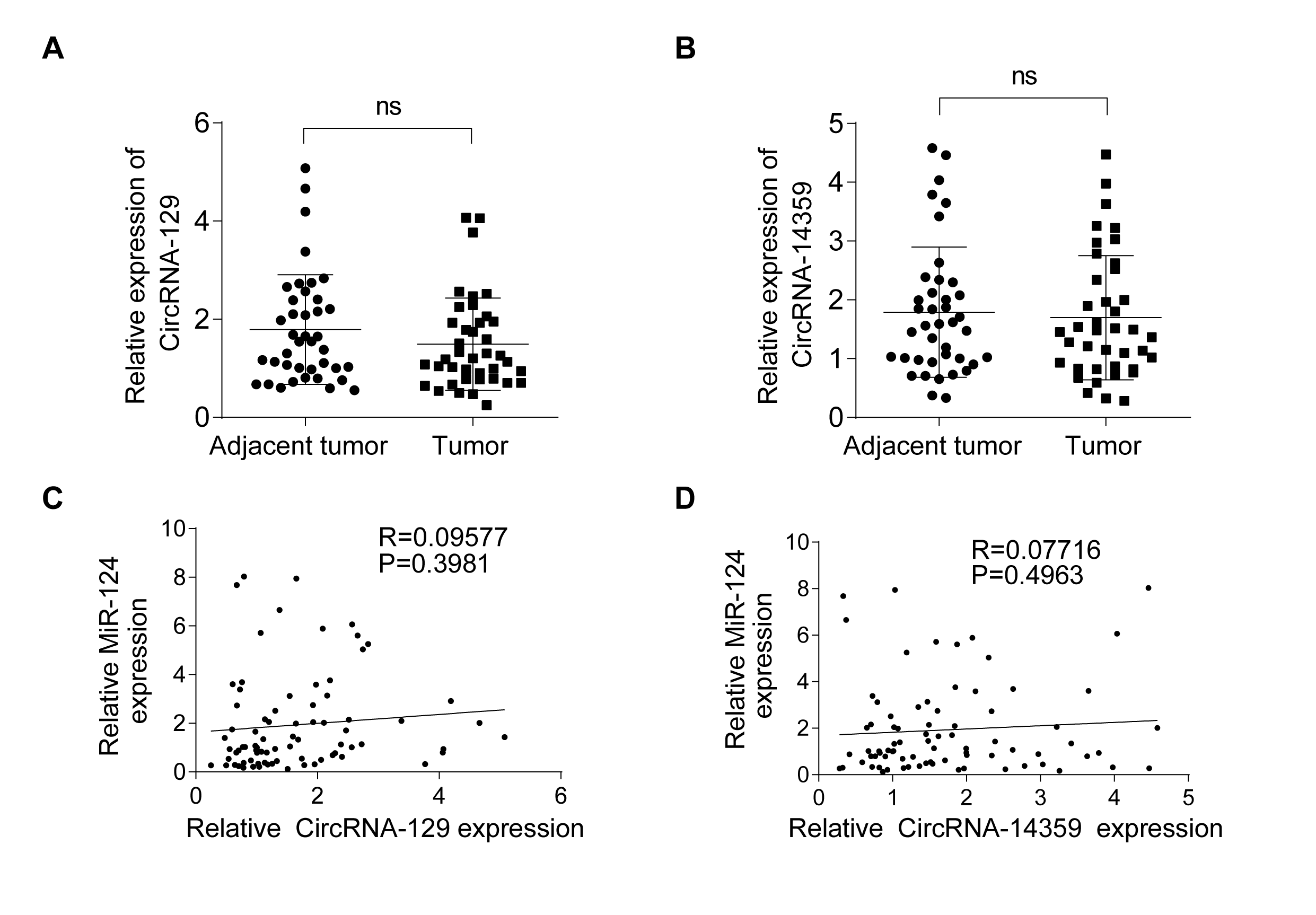

Supplement: Supplementary file 2 — The expression of CircRNA_129 and CircRNA_14359 [file 41420_2021_461_MOESM2_ESM.tif]
